# Prospective Study of Vonoprazan-Based First-Line Triple Therapy with Amoxicillin and Metronidazole for Clarithromycin-Resistant *Helicobacter pylori*

**DOI:** 10.3390/jcm12175443

**Published:** 2023-08-22

**Authors:** Soichiro Sue, Yuichi Suzuki, Tomohiko Sasaki, Hiroaki Kaneko, Kuniyasu Irie, Kazuto Komatsu, Shin Maeda

**Affiliations:** 1 Department of Gastroenterology, Yokohama City University Graduate School of Medicine, Yokohama 236-0004, Japan; ssue@yokohama-cu.ac.jp (S.S.); suzuki.yui.ar@yokohama-cu.ac.jp (Y.S.); ssktmhk@ygt-naika.com (T.S.);; 2 Department of Gastroenterology, Yokosuka City Hospital, Yokosuka 240-0195, Japan

**Keywords:** vonoprazan, metronidazole, amoxicillin, 7-day triple therapy, clarithromycin resistance, *Helicobacter pylori* eradication

## Abstract

Aim: This was a prospective, multicenter, single-arm intervention, against historical controls, study of the efficacy of a vonoprazan-based 7-day triple regimen with metronidazole (VPZ-AMPC-MNZ) as a first-line therapy for eradicating clarithromycin-resistant *Helicobacter pylori* (*H. pylori*). Methods: We enrolled 35 patients positive for clarithromycin-resistant *H. pylori*, as assessed by culture, without a history of eradication. These 35 patients were prospectively eradicated with VPZ-AMPC-MNZ. As historical controls, we also assessed 98 patients with clarithromycin-resistant *H. pylori* from our prior prospective studies, who achieved *H. pylori* eradication with a 7-day triple regimen including clarithromycin (VPZ-AMPC-CAM). A preplanned analysis was performed as a superiority study against the historical controls (VPZ-AMPC-MNZ compared to VPZ-AMPC-CAM). In each regimen, vonoprazan was used at 20 mg bid, amoxicillin at 750 mg bid, metronidazole at 250 mg bid, and clarithromycin at 200 mg or 400 mg bid for 7 days. We assessed the outcome of eradication therapy using a ^13^C-urea breath test or *H. pylori* stool antigen test. We evaluated safety using patient questionnaires. Results: The intention-to-treat (ITT) and per-protocol (PP) eradication rates of VPZ-AMPC-MNZ were both 100% (95% confidence interval (95% CI) 90.0–100%, *n* = 35). The eradication rates of VPZ-AMPC-CAM were 76.5% (95% CI 66.9–84.5%, *n* = 98) in the ITT analysis and 77.3% (95% CI 67.7–85.2%, *n* = 97) in the PP analysis. The eradication rate of VPZ-AMPC-MNZ was significantly higher than that of VPZ-AMPC-CAM in both the ITT (*p* = 0.00052) and PP (*p* = 0.00095) analyses. Conclusions: The findings suggest that 7-day VPZ-AMPC-MNZ was superior to 7-day VPZ-AMPC-CAM as a first-line regimen for eradicating clarithromycin-resistant *H. pylori*. We suggest VPZ-AMPC-MNZ as the standard first-line regimen for eradication of clarithromycin-resistant *H. pylori* in Japan.

## 1. Introduction

*Helicobacter pylori* (*H. pylori*) infection is a major risk factor for gastric cancer and peptic ulcer, and *H. pylori* eradication reduces gastric cancer incidence [1]. The World Health Organization recommends screening and treatment [2]. In Japan, *H. pylori* eradication to treat *H. pylori*-associated chronic gastritis has been covered by national health insurance since February 2013 [3]. The regimens covered are 7-day triple therapy with a proton pump inhibitor (PPI), amoxicillin (AMPC), and clarithromycin (CAM) (PPI-AMPC-CAM) as the first line, and with a PPI, AMPC, and metronidazole (MNZ) (PPI-AMPC-MNZ) as the second line [4]. Since February 2015, vonoprazan (VPZ), a potassium-competitive acid blocker (P-CAB), has been covered by national health insurance instead of a PPI [5]. Bismuth-based regimens are not approved in Japan, and sequential or concomitant regimens can be used only in clinical studies, not being covered by national health insurance [4]. Published guidelines in Japan recommend “Antimicrobials for eradication therapy should be chosen on the basis of antimicrobial susceptibility tests and used in a combination expected to achieve the highest eradication rate” [6], and published guidelines in other regions also recommend susceptibility tests of *H. pylori* and tailoring eradication regimen, which is useful for limiting the increase in antibiotic resistance by avoiding unnecessary antibiotic use [7,8,9]. In cases where individual susceptibility tests of *H. pylori* are not available, the guidelines in other regions recommend bismuth-containing quadruple therapy (PPI, bismuth, and two antibiotics), or concomitant non-bismuth quadruple therapy (PPI, and three antibiotics) [7,8,9]. However, these regimens have problems with unnecessary antibiotic use and antibiotic resistance after unsuccessful treatment. In addition, P-CAB (vonoprazan is class leader of P-CAB)-based regimens have been largely limited in East Asian countries, and P-CAB-based regimens for these regions remains unclear. In the guideline, P-CAB is expected to improve the regimens, particularly by simplifying complex regimens [9].

Because CAM- or MNZ-resistant *H. pylori* is the main cause of eradication failure [10], treatment based on antimicrobial susceptibility is desirable [11,12]. Tailored eradication therapy using PPI-AMPC-CAM for CAM-susceptible *H. pylori* and PPI-AMPC-MNZ for CAM-resistant *H. pylori* was superior to empirical PPI-AMPC-CAM therapy in an intention-to-treat (ITT) analysis (94.3% (95% CI 80.8–99.3, *n* = 35) vs. 71.4% (95% CI 53.7–85.4, *n* = 35)) [13]. Based on the high CAM resistance rate and low MNZ resistance rate in Japan [5,14], PPI-AMPC-MNZ was superior to PPI-AMPC-CAM in two randomized controlled trials (RCTs) [15,16]. The eradication rate of PPI-AMPC-CAM is unacceptable, and the 2016 Japanese guideline for the management of *H. pylori* infection (unpublished in English) recommended PPI-AMPC-MNZ as the first line; this regimen has an eradication rate of over 90% or 95% [15,16]. Regarding VPZ-containing regimens, 7-day VPZ-based triple therapy with AMPC and CAM (VPZ-AMPC-CAM) has an unacceptably low eradication rate against CAM-resistant *H. pylori*, albeit higher than that of PPI-AMPC-CAM [17,18]. Therefore, evaluating VPZ-AMPC-CAM or PPI-AMPC-CAM in patients with CAM-resistant *H. pylori* is unethical. There is little evidence for the efficacy of 7-day triple therapy with VPZ, AMPC, and MNZ (VPZ-AMPC-MNZ) as the first-line regimen, but its usefulness as a second-line therapy has been reported [19]. We recently reviewed eradication rates of VPZ-AMPC-MNZ compared to other PPI-AMPC-MNZs as second-line in Japan [20], and we showed VPZ-based treatments were slightly (~2.6%) better than PPI-based treatments, but most studies (12/13) lacked metronidazole resistance information, and most studies (11/13) were retrospective studies. First-line use of VPZ-AMPC-MNZ and PPI-AMPC-MNZ are both not covered by national insurance and are now difficult to use in Japan other than in clinical studies. There is a need for evidence of the efficacy of first-line VPZ-AMPC-MNZ for CAM-resistant *H. pylori* to enable application for coverage by national insurance. Whether VPZ-AMPC-MNZ is superior to VPZ-AMPC-CAM is thus an important question.

We evaluated the efficacy and safety of VPZ-AMPC-MNZ as the first-line regimen for eradication of CAM-resistant *H. pylori* compared to VPZ-AMPC-CAM.

## 2. Materials and Methods

### 2.1. Study Design

This was a multicenter, open-label, single-arm intervention study comparing the efficacy of eradication regimens between an intervention group (VPZ-AMPC-MNZ) and historical control group (VPZ-AMPC-CAM). The ethical reason why we did not conduct this study as an RCT design with VPZ-AMPC-CAM intervention is because the eradication rate with VPZ-AMPC-CAM for CAM-resistant *H. pylori* is reported as unacceptably low [17,18]. In this content, we thought clinical equipoise is not possible between VPZ-AMPC-MNZ for CAM-resistant *H. pylori* and VPZ-AMPC-CAM for CAM-resistant *H. pylori.* This study was conducted in accordance with the *Ethical Guidelines for Medical and Health Research Involving Human Subjects* and the Declaration of Helsinki. The study sites were Yokohama City University Hospital and Yokosuka City Hospital. This study was approved by the institutional review board of each participating hospital and was registered with the University Hospital Medical Information Network Clinical Trial Registry (https://center6.umin.ac.jp/cgi-open-bin/ctr/ctr_view.cgi?recptno=R000026412 (accessed on 15 August 2023)) as UMIN000022920 in June 2016.

### 2.2. Study Population

The inclusion criteria were male and female adult patients (age > 20 years) with CAM-resistant *H. pylori*, defined as *H. pylori* culture positive and a minimum inhibitory concentration (MIC) of CAM of ≥1.0 mg/L, based on a previous report [21], which is same as our previous studies used for historical control in this study [14,18]. *H. pylori* culture and susceptibility testing in Yokohama City University Hospital were performed by a clinical inspection agency before study enrollment; in all cases, clinical information was blinded to the agency as was the case in our previous study [18]. At the Yokosuka City Hospital, *H. pylori* culture and susceptibility testing were performed in the hospital inspection department. At both sites, gastric biopsy specimens were collected during endoscopy and transported with *H. pylori* transport medium. All culture and susceptibility testing for *H. pylori* was performed independent from the researcher and performed before study recruitment. Culture was performed with blood agar medium for *H. pylori* with addition of 10% CO_2_ at 35° urease test positive. Agar dilution tests for determining MIC were performed in accordance with National Committee for Clinical Laboratory Standards (NCCLS) Guideline M100-S9. The MIC breakpoint was set at 0.5 mg/L for AMPC [21], 8 mg/L for MNZ [21], and 0.12 mg/L for sitafloxacin (STFX) [22], based on a previous report.

If *H. pylori* infection was suspected during esophagogastroduodenoscopy, *H. pylori* culture and susceptibility tests were performed. Patients with CAM-resistant *H. pylori* infection were invited to participate this study.

Patients with any of the following were excluded from this study: pregnancy or lactation; history of *H. pylori* eradication therapy; history of allergy to the drugs used in this study; infectious mononucleosis; severe liver dysfunction; severe renal dysfunction; severe heart dysfunction; brain and/or spinal cord disease; and disqualification by a physician. Written informed consent was obtained from all of the participating patients.

### 2.3. Procedure

At the start of the study, characteristics of the patients were recorded, including age, sex, smoking, endoscopic findings, and *H. pylori* culture and susceptibility testing results. After eligibility was confirmed, including criteria and written informed consent, patients were assigned to study regimen (see Treatment section). After the administration of study regimen, physical assessment was performed by physician. Treatment compliance was confirmed by physician. Adverse effects questionnaire (AEQ) (see Safety Assessment section) was also collected. UBT or *H. pylori* stool antigen test were performed for the eradication assessment at least 6 weeks after study regimen had finished.

### 2.4. Treatment

Registered patients were assigned to receive the following regimen: triple therapy with vonoprazan (VPZ) 20 mg, amoxicillin (AMPC) 750 mg, metronidazole (MNZ) 250 mg, twice daily for 7 days (VPZ-AMPC-MNZ). The patients in the historical control group received VPZ 20 mg, AMPC 750 mg, and clarithromycin (CAM) 200 mg or 400 mg twice daily for 7 days (VPZ-AMPC-CAM).

### 2.5. Outcome

We hypothesized that VPZ-AMPC-MNZ would be superior to VPZ-AMPC-CAM as a first-line regimen for eradication of CAM-resistant *H. pylori*.

The primary endpoint was the eradication rate of *H. pylori* assessed using a ^13^C-urea breath test (UBT) or *H. pylori* stool antigen test after the end of first-line therapy. We compared the eradication rate of VPZ-AMPC-MNZ and VPZ-AMPC-CAM.

All patients were asked to stop vonoprazan, PPIs, histamine-2 blockers, and antibiotics for at least 4 weeks before undergoing a UBT or *H. pylori* stool antigen test, because both tests are accepted evaluation methods of *H. pylori* eradication success [23,24]. UBT was performed using UBIT (100 mg) tablets (Otsuka Pharmaceutical). The UBT or *H. pylori* stool antigen test was performed by an external clinical inspection agency or by the internal inspection units of the participating hospitals; in all cases, the operators were blinded to the group assignment.

We performed ITT and per-protocol (PP) primary analyses. The ITT analysis included all patients who started the eradication therapy. The patients who did not undergo UBT or *H. pylori* stool antigen test after eradication, or who were lost to follow-up, were treated as eradication failures in the ITT analysis. Eradication success was defined as <2.5‰ in a UBT or a negative *H. pylori* stool antigen test.

### 2.6. Safety Assessment

The secondary endpoint was safety as evaluated using an adverse effects questionnaire (AEQ) completed by the patient, and the same AEQ was used between groups [14,18]. The AEQ comprises questions on diarrhea, dysgeusia, nausea, anorexia, abdominal pain, heartburn, urticaria, headache, abdominal fullness, eructation, vomiting, fatigue, and others. The possible responses were none (AEQ 0), weak (AEQ 1), moderate (AEQ 2), or strong (AEQ 3). Completed AEQs were collected at the start of the clinical examination, and there was no reporting bias.

### 2.7. Statistical Analysis

We assumed that the eradication success rate of VPZ-AMPC-MNZ for CAM-resistant *H. pylori* was 98.0% (95% confidence interval (CI) 89.4–99.9%), according to a phase III trial of VPZ as a second-line regimen [5]. We also assumed an eradication success rate of VPZ-AMPC-CAM for CAM-resistant *H. pylori* of 75% [14,18]. We set the statistical power at 80% (1-β), and the significance level by two-sided test at 5%, with an assumed loss to follow-up of 10%. We set the sample size at 35 patients who received VPZ-AMPC-MNZ.

Fisher’s exact test and Student’s *t*-test were applied to categorical and continuous data, respectively, with a significance level of 5% by two-sided test. Frequencies and two-sided 95% CIs were calculated for the primary endpoint. Fisher’s exact test was performed to evaluate the difference in the eradication rates of VPZ-AMPC-MNZ and VPZ-AMPC-CAM for CAM-resistant *H. pylori*. Statistical analysis was performed using SPSS software (ver. 25.0) (IBM, Armonk, NY, USA).

## 3. Results

### 3.1. Study Flow

**Figure 1:**
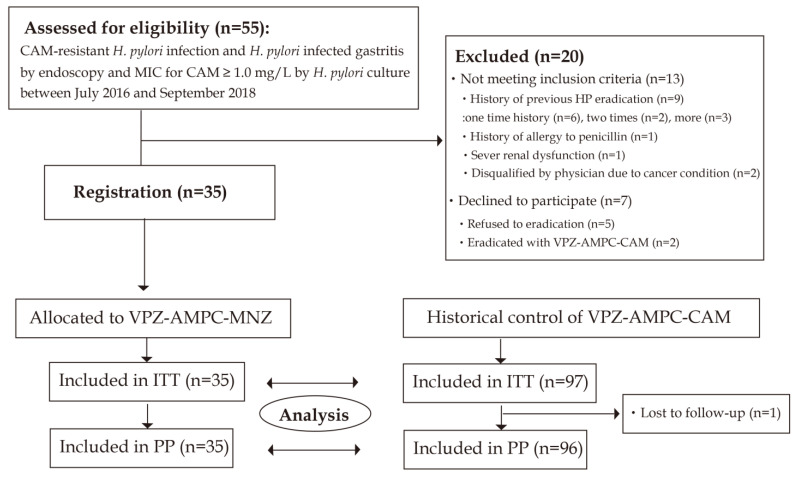
Flow chart of patient enrollment. ITT: intention-to-treat; PP: per protocol; VPZ-AMPC-MNZ: 7-day triple therapy with vonoprazan, amoxicillin, and metronidazole; VPZ-AMPC-CAM: 7-day triple therapy with vonoprazan, amoxicillin, and clarithromycin.

The study flow is summarized in Figure 1. Between July 2016 and September 2018, patients were enrolled. A total of 55 patients who were diagnosed as having CAM-resistant *H. pylori* infection and *H. pylori*-infected gastritis using endoscopy and MIC for CAM ≥ 1.0 mg/L using *H. pylori* culture were assessed for eligibility. Twenty patients were excluded because they did not meet the inclusion criteria (*n* = 13) or declined to participate (*n* = 7). As a result, 35 patients provided written informed consent prior to study participation and registered to the study. Thirty-three patients were assessed using UBT and two patients were assessed using an *H. pylori* stool antigen test. UBT or *H. pylori* stool antigen tests were conducted 11.4 ± 8.0 weeks (minimum 6.7 weeks) after the VPZ-AMPC-MNZ eradication therapy finished. The last follow-up date was December 2018. Thirty-five patients with CAM-resistant *H. pylori* infection without a history of eradication were enrolled and received VPA-AMPC-MNZ.

Next, we compared our results with those of 98 patients in whom CAM-resistant *H. pylori* infection was eradicated using first-line VPZ-AMPC-CAM as historical control (56 from a prospective multicenter cohort study [14] and 41 from a multicenter intervention study [18]). One patient was lost to follow-up; the other 97 cases were subjected to evaluation of the *H. pylori* eradication success rate. Registration and follow-up period were from February 2015 to February 2016 [14] and from February 2015 to October 2016 [18]. Ninety-seven patients were all assessed using UBT.

### 3.2. Baseline Characteristics

Baseline characteristics are listed in Table 1. The patients in the intervention and historical control groups were similar in terms of age (VPZ-AMPC-MNZ: 62.3 ± 13.7 years, VPZ-AMPC-CAM: 64.8 ± 12.7 years) but not in sex distribution (VPZ-AMPC-MNZ: male 71.4%, VPZ-AMPC-CAM: male 45.9%). The smoking rate (VPZ-AMPC-MNZ: 20.0%, VPZ-AMPC-CAM: 14.3%), and endoscopic findings were similar in the two groups. Endoscopic findings between groups were comparable, and most cases were Gastritis only. All patients in this study confirmed *H. pylori* infection with *H. pylori* culture. All patients in both groups were infected with AMPC-susceptible *H. pylori*. In the VPZ-AMPC-MNZ group, the MNZ resistance rate was 3.4% and that of STFX resistance was 37.9%. (Table 1).

### 3.3. Efficacy

**Figure 2:**
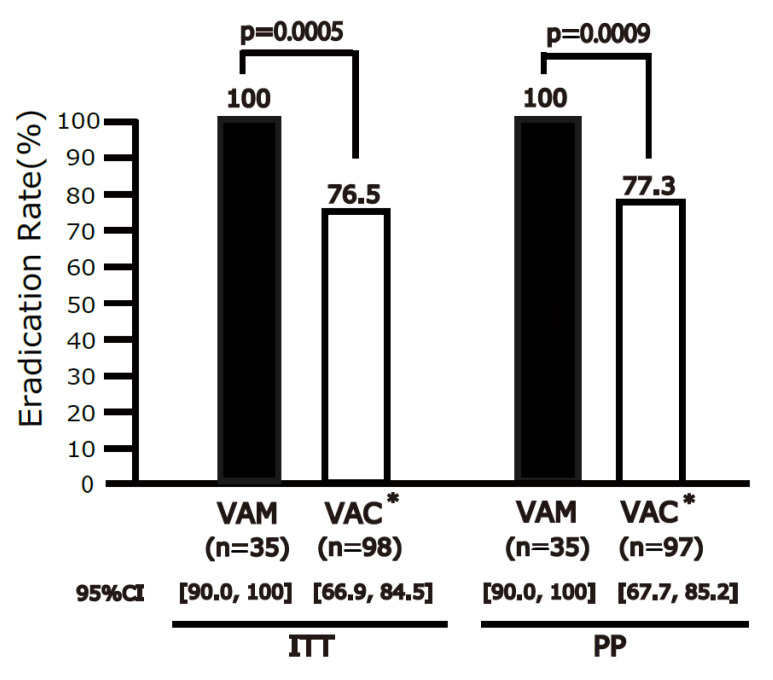
Eradication rates of VPZ-AMPC-MNZ and VPZ-AMPC-CAM in intention-to-treat (ITT) and per-protocol (PP) analyses. The eradication rate differed significantly between the regimens in both the ITT and PP analyses. VPZ-AMPC-MNZ: 1-week triple therapy with vonoprazan, amoxicillin, and metronidazole; VPZ-AMPC-CAM: 1-week triple therapy with vonoprazan, amoxicillin, and clarithromycin. * VPZ-AMPC-CAM arm constitutes the historical controls from our two prior studies [14,18].

The eradication rate was 100% (95% CI 90.0–100%, *n* = 35) for VPZ-AMPC-MNZ and 76.5% (95% CI 66.9–84.5%, *n* = 98) for VPZ-AMPC-CAM in the ITT analysis (Figure 2). In the PP analysis, the eradication rate was 100% (95% CI 90.0–100%, *n* = 35) for VPZ-AMPC-MNZ and 77.3% (95% CI 67.7–85.2%, *n* = 97) for VPZ-AMPC-CAM. VPZ-AMPC-MNZ was superior to VPZ-AMPC-CAM in both the ITT (*p* = 0.0005) and PP (*p* = 0.0009) analyses.

### 3.4. Safety and Compliance

The frequencies of adverse effects during therapy are shown in Table 2. The frequency of any symptoms (score 1, 2, or 3) of diarrhea, dysgeusia, nausea, anorexia, abdominal pain, heartburn, hives, headache, abdominal fullness, belching, vomiting, general malaise, or other was not significantly different between VPZ-AMPC-MNZ and VPZ-AMPC-CAM for CAM-resistant *H. pylori*. The rate of a score of 3 for anorexia was significantly higher for VPZ-AMPC-MNZ (9%) than VPZ-AMPC-CAM (0%). All adverse events spontaneously resolved without intervention and no patient was hospitalized due to adverse events. All patients in both groups received 100% of the prescribed regimen.

## 4. Discussion

This is, to our knowledge, the first registered prospective study to show the superiority of VPZ-AMPC-MNZ over VPZ-AMPC-CAM as the first-line eradication regimen for CAM-resistant *H. pylori*. VPZ-AMPC-MNZ was categorized as good or excellent for CAM-resistant *H. pylori* based on the 95% CI of 90–100%.

It is thought that it is difficult for registered prospective studies to be affected by publication bias and other biases. Most prior studies of VPZ-based eradication were of retrospective design, which could be misleading, especially in the absence of information on the antibiotic resistance of *H. pylori*.

We used an AEQ to avoid evaluator bias when evaluating adverse events. The rate of a score of 3 for anorexia was significantly higher for VPZ-AMPC-MNZ (9%) than VPZ-AMPC-CAM (0%). All symptoms resolved spontaneously, and compliance was good. Our findings indicate that a first-line VPZ-AMPC-MNZ regimen has a safety profile similar to that of second-line VPZ-AMPC-MNZ for eradication of *H. pylori*.

In the setting of AMPC susceptibility (100%) and 96.6% susceptible to MNZ *H. pylori* infection, the VPZ-AMPC-MNZ regimen was optimal (ITT, 95–100%) or acceptable (ITT, 90–95%) as the first-line CAM-resistant *H. pylori* eradication regimen (Table 1). In Japan, the CAM resistance rate is >30% compared to <5% for MNZ resistance, and a negligible rate for AMPC resistance using breakpoints of 1 mg/L for CAM, 0.5 mg/L for AMPC, and 8 mg/L for MNZ. Therefore, this study setting can be generalizable from the perspective of the antibiotic resistance background of *H. pylori* in Japan. Only two prospective studies have evaluated VPZ-AMPC-MNZ [5, 14], one of which provided resistance information [5]. Murakami reported an eradication rate of 98% (95% CI 89.4–99.9%, *n* = 50) for 90% MNZ-susceptible *H. pylori*, and we found a rate of 80.8% (95% CI 74.6–85.6%, *n* = 216) as a second-line regimen, but resistance information was lacking. This is the first registered prospective study of VPZ-AMPC-MNZ as a first-line *H. pylori* eradication regimen. To compare the first- and second-line results, we must consider selection bias, because most patients in the second-line study failed to eradicate *H. pylori* with the first-line VPZ-AMPC-CAM regimen. This registered prospective study is valuable in that it demonstrates the superiority of VPZ-AMPC-MNZ to VPZ-AMPC-CAM as the first-line eradication regimen for CAM-resistant *H. pylori*.

VPZ is potassium-competitive acid blocker that shows rapid (pH > 4.0 within 4 h), strong (pH > 5.0 for 99% of the time at 20 mg bid), and stable (unaffected by the CP2C19 genotype) H+/K+ ATPase inhibition. A VPZ-AMPC first-line regimen had an eradication rate of 84.5% (95% CI 78.2–89.6%) and 92.3% (95% CI 79.1–98.4%) for CAM-resistant *H. pylori* [25]. Dong et al. reported that VPZ-AMPC-CAM is superior to PPI-AMPC-CAM for CAM-resistant *H. pylori* (eradication rate 81.5% (95% CI 75.0–86.9%) vs. 40.9% (95% CI 34.4–47.6%)) [15]. We reported that VPZ-AMPC-STFX has a higher eradication rate than PPI-AMPC-STFX as the third-line regimen [26]. Recently, VPZ and AMPC dual therapy studies from several regions were reviewed. Regimen periods were 7-day, 10-day, or 14-day periods and AMPC doses were 500 mg tid, 750mg bid, 500 mg qid, 750 mg qid, 1000 mg bid, 750 mg qid, or 1000 mg tid. The eradication rates were from 58.3% to 95.3% [27]. These results suggest the efficacy of VPZ-based regimens, but VPZ-AMPC and VPZ-AMPC-CAM did not achieve good (90–95%) or excellent (95–100%) eradication rates for CAM-resistant *H. pylori*. In summary, we think the reason why all were successfully eradicated in this study is based on no AMPC resistance and low MNZ resistance rates and the efficacy of VPZ.

We published two registered prospective studies, in which the VPZ-AMPC-CAM eradication rate for CAM-resistant *H. pylori* was 73.2% (95% CI 59.7–84.2%) [14] and 82.9% (95% CI 67.9–92.8%, *n* = 41) [18]. From these results, we concluded that VPZ-based regimens, although useful, have insufficient efficacy against CAM-resistant *H. pylori* and so conducted a single-arm intervention study using the VPZ-AMPC-CAM results as historical controls. Our findings suggest that VPZ-AMPC-MNZ should be used as the first-line eradication regimen for CAM-resistant *H. pylori* instead of VPZ-AMPC-CAM, particularly for *H. pylori* susceptible to AMPC and MNZ.

We used historical control of 77.3% (95% CI 67.7–85.2%, *n* = 97) with VPZ-AMPC-CAM for *H. pylori* with CAM-resistance as first line. These data are consistent with the two prospective [5,25] and two retrospective [28,29] studies of VPZ-AMPC-CAM for CAM-resistant *H. pylori*. Murakami reported an eradication rate of 82% (95% CI 73.1–89.0%, *n* = 100), compared to 76.2% (95% CI 60.5–87.9%, *n* = 42) by Suzuki, 87.5% (95% CI 71.0–96.5%, *n* = 32) by Noda, and 76.1% (95% CI 61.2–87.4%, *n* = 46) by Matsumoto. None of those studies achieved a good (90–95%) or excellent (95–100%) success rate.

VPZ-AMPC-MNZ is suitable as the first-line regimen for AMPC- and MNZ-susceptible *H. pylori*. In Japan, empiric first-line treatment with VPZ-AMPC-MNZ may be useful. However, in other countries with a high MNZ-resistance rate, VPZ-AMPC-MNZ should be used for AMPC- and MNZ-susceptible *H. pylori* infection.

In an unregistered retrospective study, VPZ-AMPC-MNZ for CAM-resistant *H. pylori* had a 98.5% eradication rate (95% CI 86.2–95.7%, *n* = 147) in an ITT analysis and a 91.8% eradication rate (95% CI 94.8–99.8%, *n* = 137) in a PP analysis. [30] The MNZ resistance rate was 1.4%, but the AMPC resistance rate was not reported. Our findings are in agreement with these results.

Unexpectedly, we found a 37.9% STFX-resistance rate in CAM-resistant, AMPC-susceptible and slightly MNZ-resistant *H. pylori*. Therefore, VPZ-AMPC-STFX is a good rescue regimen if first-line VPZ-AMPC-MNZ fails to eradicate CAM-resistant but STFX-susceptible *H. pylori*. Further investigation of VPZ-AMPC-STFX as second-line for first-line VPZ-AMPC-MNZ failure is needed. This result suggests the importance of resistance information for CAM, AMPC, MNZ, and STFX. In this study, limited facilities were used to conduct all antibiotic susceptibility tests of AMPC, CAM, MNZ, and STFX.

To confirm this study’s result, further registered prospective studies of first-line VPZ-AMPC-MNZ for CAM-resistant *H. pylori* is desirable.

This study had several limitations. First, its nonrandomized controlled design, using historical controls, was selected because of ethical concerns over an RCT. The main confounder affecting the eradication rate was antibiotic resistance; the difference in gender distribution between the groups is unlikely to have affected the results. The rate of CAM-resistant *H. pylori* infection is higher in females [30], but this study included all patients with CAM-resistant *H. pylori*. Second, sample size of this study was small. This pre-planned sample size calculation was based on high expected eradication rate based on previous study, as written in the statistical analysis section.

## 5. Conclusions

In summary, VPZ-AMPC-MNZ was superior to VPZ-AMPC-CAM for CAM-resistant *H. pylori* first-line eradication. We suggest VPZ-AMPC-MNZ as the standard regimen for CAM-resistant *H. pylori* infection in the setting of low AMPC and MNZ resistance rates. Our findings indicate that, in the absence of antibiotic resistance information, VPZ-AMPC-MNZ should be the first-line empiric regimen in Japan, where the rate of CAM resistance is high but that of AMPC and MNZ resistance is low.

## Figures and Tables

**Table 1:** Baseline characteristics.

	VPZ-AMPC-MNZ	VPZ-AMPC-CAM *	*p*
Age	62.3 ± 13.7	64.8 ± 12.7	0.35
Male, %	71.4	45.9	0.01
Smoking, %	20.0	14.3	0.43
Endoscopic findings, %			
Gastroduodenal ulcer	5.7	4.0	
Gastric cancer	2.9	1.0	0.51
Gastritis only	91.4	95.0	
Diagnosis of infection, %			
Culture	100	100	1
CAM resistance, %	100 (35/35)	100 (98/98)	1
AMPC resistance, %	0 (0/35)	0 (0/98)	1
MNZ resistance, %	3.4 (1/29)	N/A	
STFX resistance, %	37.9 (11/29)	N/A	

VPZ-AMPC-MNZ: 1-week triple therapy with vonoprazan, amoxicillin, and metronidazole; VPZ-AMPC-CAM: 1-week triple therapy with vonoprazan, amoxicillin, and clarithromycin; UBT: urea breath test; culture: *Helicobacter pylori* culture; N/A: not available. * VPZ-AMPC-CAM arm constitutes the historical controls from our two prior studies [14,18].

**Table 2:** Adverse effects.

**Any (Score 1, 2 or 3)**	**VPZ-AMPC-MNZ**	VPZ-AMPC-CAM *	***p***
Diarrhea	34%	16%	0.07
Dysgeusia	19%	15%	0.77
Nausea	16%	8%	0.31
Anorexia	19%	10%	0.33
Abdominal pain	13%	10%	0.73
Heartburn	16%	16%	1
Hives	0%	2%	1
Headache	3%	16%	0.09
Abdominal fullness	31%	30%	1
Belch	22%	13%	0.38
Vomiting	0%	0%	1
General malaise	16%	12%	0.75
Other	13%	5%	0.23
**Score 3**	**VPZ-AMPC-MNZ**	VPZ-AMPC-CAM *	***p***
Diarrhea	0%	0%	1
Dysgeusia	0%	8%	0.16
Nausea	0%	0%	1
Anorexia	9%	0%	0.04
Abdominal pain	0%	0%	1
Heartburn	0%	3%	0.54
Hives	0%	3%	0.54
Headache	0%	0%	1
Abdominal fullness	9%	10%	1
Belch	6%	8%	1
Vomiting	0%	0%	1
General malaise	3%	0%	0.35
Other	0%	0%	1

VPZ-AMPC-MNZ: 1-week triple therapy with vonoprazan, amoxicillin, and metronidazole; VPZ-AMPC-CAM: 1-week triple therapy with vonoprazan, amoxicillin, and clarithromycin. * VPZ-AMPC-CAM arm is the historical controls from our two prior studies [14,18].

## Data Availability

The data are not available for public access because of ethical restrictions but are available from the corresponding author on reasonable request.
